# Sensory modulation dysfunction is associated with Complex Regional Pain Syndrome

**DOI:** 10.1371/journal.pone.0201354

**Published:** 2018-08-09

**Authors:** Tami Bar-Shalita, Anatoly Livshitz, Yulia Levin-Meltz, Debbie Rand, Lisa Deutsch, Jean-Jacques Vatine

**Affiliations:** 1 Department of Occupational Therapy, School of Health Professions, Sackler Faculty of Medicine, Tel Aviv University, Tel Aviv, Israel; 2 Sagol School of Neuroscience, Tel Aviv University, Tel Aviv, Israel; 3 Center for Rehabilitation of Pain Syndromes, Reuth Rehabilitation Hospital, Tel Aviv, Israel; 4 BioStats Statistical Consulting Ltd, Modiin, Israel; 5 Sackler Faculty of Medicine, Tel Aviv University, Tel Aviv, Israel; BG-Universitatsklinikum Bergmannsheil, Ruhr-Universitat Bochum, GERMANY

## Abstract

**Objective:**

Complex Regional Pain Syndrome (CRPS), a chronic pain condition, develops mainly after limb trauma and severely inhibits function. While early diagnosis is essential, factors for CRPS onset are elusive. Therefore, identifying those at risk is crucial. Sensory modulation dysfunction (SMD), affects the capacity to regulate responses to sensory input in a graded and adaptive manner and was found associated with hyperalgesia in otherwise healthy individuals, suggestive of altered pain processing.

**Aim:**

To test SMD as a potential risk factor for CRPS.

**Methods:**

In this cross-sectional study, forty-four individuals with CRPS (29.9±11 years, 27 men) and 204 healthy controls (27.4±3.7 years, 105 men) completed the Sensory Responsiveness Questionnaire-Intensity Scale (SRQ-IS). A physician conducted the CRPS Severity Score (CSS), testing individuals with CRPS.

**Results:**

Thirty-four percent of the individuals with CRPS and twelve percent of the healthy individuals were identified to have SMD (χ2 (1) = 11.95; *p*<0.001). Logistic regression modeling revealed that the risk of CRPS is 2.68 and 8.21 times higher in individuals with sensory over- and sensory under-responsiveness, respectively, compared to non-SMD individuals (*p* = 0.03 and *p* = 0.01, respectively).

**Conclusions:**

SMD, particularly sensory under-responsiveness, might serve as a potential risk factor for CRPS and therefore screening for SMD is recommended. This study provides the *risk index probability clinical tool* a simple evaluation to be applied by clinicians in order to identify those at risk for CRPS immediately after injury. Further research is needed.

## 1. Introduction

Complex Regional Pain Syndrome (CRPS) develops in 4–7% of patients after limb fractures, limb surgery or other injuries [1, 2] and is characterized by continuing (evoked and/or spontaneous) pain that is regional related, disproportionate in its time duration and/or pain intensity after trauma or other lesions. Further, the pain typically has a distal predominance of abnormal sensory, motor, sudomotor, vasomotor edema, and/or trophic characteristics [3, 4]. Though the evolution of CRPS varies, it usually evolves into severe state of disablement in the affected limb, which adversely impacts function and quality of life [3, 5, 6]. Consequently, the individual burden that CRPS enforces and the costs that society has to bear are vast; patients with CRPS who have prolonged pain, typically reduce working hours or are unemployed, thus salary income is usually decreased [4, 7] while overall costs are elevated [7]. The incidence of CRPS in Europe per year is estimated to be about 26.2 per 100,000 individuals [8, 9] and approximately 200,000 Americans are diagnosed with CRPS every year [2]. Total annual income loss in the US due to CRPS exceeds US$1 billion [10].

There are two types of CRPS; CRPS that involves distinct nerve injury (Type 2), and CRPS where there is no identifiable nerve injury (Type 1; identified by a noxious event or a cause of immobilization) [11]. The etiology of CRPS is still unclear and several underlying pathophysiological mechanisms have been indicated. These consist of genetic and psychological factors; somatosensory innervation alterations; peripheral and central sensitization; altered sympathetic nervous system processing; augmented levels of local and systemic inflammatory cytokines; lower systemic levels of anti-inflammatory cytokines; lower levels of circulating catecholamines; and regulating neuroplasticity difficulties [12–14]. Early diagnosis of CRPS and prompt rehabilitation is widely recommended as the best way to achieve optimum outcomes [11, 15, 16]. However, failure to consider complicating factors delays the diagnosis, and improper treatment can lead to CRPS with severe disability [8].

Different potential risk factors for the onset of CRPS have been suggested. Risk is higher in females, mostly postmenopausal women; individuals who have suffered an ankle injury including dislocation or an intraarticular fracture, and a wrist fracture; those who are immobilized; and when the pain level is enhanced, higher than usual, in early stages of post trauma [11, 17]. Furthermore, a significant association between CRPS and a prior diagnosis of migraine or osteoporosis has been reported [18] although these potential risk factors were not confirmed across trials [11, 17]. Evidence for robust genetic associations has not been identified [11, 17], and psychological factors such as depression, neuroticism, anxiety or anger have also not been shown to be predictors for CRPS development [19]. Recently however, higher prevalence of posttraumatic stress disorder (PTSD) in CRPS, compared to the general population and to other limb pain disorders, was reported indicating that PTSD may serve as a risk factor for developing CRPS [20]. Importantly, while factors for CRPS onset are still elusive [11], identifying those at risk for CRPS prior to surgical interventions, immediate post-trauma [1], or very early in the condition, is warranted [3, 6].

A distinguished body of literature about CRPS supports the involvement of the central nervous system in terms of sensory as well as pain processing changes [8, 21, 22]. Specifically, not only have impairments in endogenous pain inhibitory pathways evident, but these were associated with pain severity [23, 24], showing pain hyperalgesia [25]. Moreover, reported sensory changes in the contralateral hand were found associated with the ones occurring in the affected hand, [26], indicating central sensory processing alterations as well. To-date it is not clear whether these changes are a consequence of or a predisposition for developing CRPS [23].

Sensory modulation dysfunction (SMD), is a type of sensory processing disorder, impacting single or multiple sensory systems, affecting the capacity to regulate responses to sensory input in a graded and adaptive manner [27]. SMD greatly limits and interferes with quality of life, work performance and participation in everyday routines [28–31] and its prevalence is estimated to be 5–16% of the population, otherwise healthy [28, 32, 33]. SMD is characterized by: (i) sensory over-responsiveness in which non-painful stimuli are perceived as abnormally unpleasant, aversive [27, 34–39], or painful [37, 40–43], and/or (ii) sensory under-responsiveness demonstrated by reduced responses to stimuli [27, 29, 32, 34–38, 42]. Sensory over-responsiveness has been found to be associated with daily pain sensitivity [28]. Moreover, laboratory quantitative sensory testing has revealed pain perception alterations in individuals with sensory over-responsiveness; Children and adults with sensory over-responsiveness have demonstrated pain hyper-sensitivity (hyperalgesia) and intense prolonged duration of pain lingering sensations, compared to controls [40, 41], suggesting compromised endogenous pain modulation in individuals with sensory over-responsiveness who are otherwise healthy [43].

This study aims to explore the association between CRPS and SMD, specifically to examine whether SMD could be considered as a risk factor for CRPS. Such a simple and efficient measure to identify a potential risk for CRPS may encourage an earlier diagnosis, improve the prognosis, and dramatically reduce the societal load [8].

## 2. Materials and methods

This cross-sectional study included a two-group comparison between participants with and without CRPS.

### 2.1 Participants

Participants with CRPS were recruited from the database of the Center for Rehabilitation of Pain Syndromes, Reuth Rehabilitation Hospital. Inclusion criteria stipulated participants were to be at least 18 years of age and experiencing pain for at least 3 months. CRPS diagnosis was determined by a specialist physician in physical medicine and rehabilitation and an expert in pain management, according to the Budapest Research Criteria [1]. These criteria include continuous pain that is disproportionate to any inciting event, and a report of at least one symptom in the following categories (sensory, vasomotor, sudomotor, and motor/trophic). Furthermore, at least one sign had to be present in 2 or more of the categories (sensory, vasomotor, sudomotor, and motor/trophic) at the time of examination, without having any other condition that could account for the signs and symptoms encountered. The Budapest Research Criteria have a sensitivity of .78 and a specificity of .79 for the diagnosis of CRPS [1].

The participants without CRPS were healthy adult volunteers, henceforth the control group, who were recruited by convenience and snowball sampling. They were comprised of 48.5% university students while the rest were mainly employed individuals recruited off campus.

Exclusion criteria for both groups included pregnancy; current or history of any neurological disorder, including speech, vision, hearing or behavioral abnormalities; and the presence of a cognitive impairment or any other factor resulting in the inability to understand and/or to complete self-report questionnaires. Exclusion criterion for the study group also included pain disorder other than CRPS. Additional exclusion criteria for the control group included any pain disorder, a family history of siblings, parents, and/or grandparents that included any form of psychopathology.

The research was approved by the Reuth Rehabilitation Hospital review board, and the study protocol conformed to the ethical guidelines of the 1975 Declaration of Helsinki. All participants provided written consent before enrolling in the study.

### 2.2 Measures

#### 2.2.1 The Sensory Responsiveness Questionnaire—Intensity Scale

[44] is a standardized reliable and valid self-report questionnaire, assessing responses to daily sensations and is used to clinically identify SMD in adults [44, 45]. The Sensory Responsiveness Questionnaire—Intensity Scale is comprised of a set of 58 items representing typical scenarios occasionally occuring throughout daily life. Each item consists of one sensory stimulus in one modality including somatosensory, olfactory, gustatory, vestibular, auditory and visual stimuli (excluding pain). The items are worded in a manner attributing an aversive /hedonic valence to the scenarios (e.g. *I enjoy wearing woolen clothes on myself*). Using a 5-point scale, participants rate the intensity of the aversive /hedonic response to the scenarios, with the anchors ‘*not at all’* (1) to *‘very much’* (5). The Sensory Responsiveness Questionnaire—Intensity Scale has been demonstrated to have an internal consistency (Cronbach α = 0.90–0.93) and test-retest reliability (r = 0.71–0.84; *p* < 0.001–0.005) as well as content, construct and criterion validity [44].

In this study, sensory over-responsiveness SOR sub-type was determined by applying the Sensory Responsiveness Questionnaire—Intensity Scale—Aversive sub-score (32 items), in which scores were 2 standard deviations above the mean cut-off score (1.87 **+** 0.52) for normal responsiveness. Sensory under-responsiveness sub-type was determined applying the Sensory Responsiveness Questionnaire—Intensity Scale—Hedonic sub-score (26 items), for which scores were above the mean cut-off score +2SD (2.10 **+** 0.66) for normal responsiveness. Participants scoring higher than one or both cut-off scores comprised the SMD group. A mean+2 SD cut-off score was used to ensure cautious estimation of the SMD prevalence.

#### 2.2.2 The CRPS Severity Score (CSS)

[46], is a standard score which provides a continuous type quantitative index of the CRPS signs and symptom severity as they are identified by current diagnostic criteria. CRPS diagnosis based on the Budapest criteria is a dichotomous (yes/no) diagnostic decision [1], and does not provide information of individual clinical presentation differences, severity, or liability of CRPS signs and symptoms. The CRPS Severity Score comprises 17 items of the Budapest criteria (8 self-reported symptoms and 9 signs observed on examination by a clinician). Each present sign/symptom is counted as one point and added to a total score, ranging from 0 to 17, which constitutes the CRPS Severity Score score [1]. Higher scores indicate greater CRPS severity. The CRPS Severity Score has been shown to have high internal consistency, discriminate validity, and strong association with both the current International Association for the Study of Pain diagnostic criteria and the proposed Budapest criteria [46].

### 2.3 Procedure

Participants were contacted by phone, and the study’s purpose and other preliminary information were provided by the researcher while exclusion criteria were verified. For participants in the CRPS group, the physician conducted an on-site evaluation using the diagnostic signs and symptoms of CRPS, permitting the calculation of the CSS [1]. After CRPS diagnosis was confirmed, subjects were asked to complete a medical and demographic questionnaire as well as the Sensory Responsiveness Questionnaire—Intensity Scale [44]. This evaluation meeting lasted for approximately one hour. As for the control group, participants met with the researcher and completed a medical and demographic questionnaire followed by the Sensory Responsiveness Questionnaire—Intensity Scale. This session lasted for approximately 30 minutes, while the researcher was present and available for participants’ queries.

### 2.4 Data analysis

Statistical analyses were performed with SAS V9.3 (SAS Institute, Cary NC, USA). Continuous variables were summarized by the mean and standard deviation and compared with a t-test, ANOVA or a non-parametric equivalent. Categorical variables were summarized by count and percentage, and compared with Fisher’s exact test. All statistical tests were two-sided and tested at a 5% level of significance. This is an exploratory study since previous data between SMD and CRPS does not exist, thus nominal p-values were presented, without a correction for multiple testing. Logistic regression modeling was performed to identify risk factors for CRPS (age, gender, SRQ scores, CRPS signs and symptoms (CCS)). Variables found to be significant (with p<0.05) were entered into a multivariate model. The variables designated to remain in the model were those risk factors of CRPS which remained statistically significant when entered together and that maximized the predictive power (area under the curve [AUC] of the receiver operating characteristic [ROC] curve) of the model, such that the AUC of the resulting ROC curve was at least 0.8. The risk score, which was calculated from a linear combination of the logistic regression model coefficients, is presented in a figure, as an effect plot, portraying the risk of having CRPS as a function of SMD sub-type and age.

## 3. Results

Two hundred and forty-eight subjects were recruited of whom 44 (17.7%) were CRPS patients (27 men, 61.4%), 3 months to 12-years post diagnosis (mean±SD, 3.18±3.16 years; median, 2.01 years). Specifically, CRPS type-I was diagnosed in 35 (79.5%) patients while 9 (20.5%) were diagnosed with CRPS type-II. Two hundred and four individuals (82.3%51.5%) served as the healthy control group (105 men, 51.5%). Sex distribution differences between groups were not found to be statistically significant (χ^2^ (1) = 1.42; *p* = 0.233). Mean age was also not found to be statistically significant between groups t(45.162) = -1.54; *p* = 0.130; 29.9±11.0 *vs*. 27.4±3.7 years in the CRPS (range: 19–61 years) and control groups (range: 23–40 years), respectively. Table 1 summarizes the site and etiology of the patients with CRPS.

**Table 1:** CRPS site and etiology (N = 44).

	Location		Etiology						Total
	Body-side		Fracture	Surgery	Crush Injury	Contusion	Tendon tear	Unidentified	n
	RT	LT							
**Hand (palm)**	2	2	1	1	2	0	0	0	4
**Wrist**	1	3	2	1	0	0	1	0	4
**Shoulder**	3	0	0	2	0	1	0	0	3
**Knee**	3	1	1	2	1	0	0	0	4
**Ankle**	4	1	2	1	2	0	0	0	5
**Foot**	13	11	8	7	6	1	1	1	24
**Total**	26	18	14	14	11	2	2	1	44

### 3.1 SMD distribution in CRPS and in healthy controls

Thirty-four percent (N = 15) of the individuals with CRPS and 12.8% (N = 26) of the healthy controls were identified with SMD. This difference was found to be statistically significant (χ^2^ (1) = 11.95; *p*<0.001). SMD sub-types distribution was also found statistically different between groups: In the CRPS group 25% (N = 11) and 9.1% (N = 4) met the criteria for sensory over-responsiveness and sensory under-responsiveness, respectively, versus the control group in which 11.3% (N = 23) and 1.5% (N = 3) met the criteria for sensory over-responsiveness and sensory under-responsiveness, respectively (χ^2^ (1) = 14.39, *p*<0.001; Table 2). Of note, no differences were found in both sensory over-responsiveness and sensory under-responsiveness scores within the CRPS group between patients with CRPS type 1 *vs*. patients with CRPS type 2 (for sensory over-responsiveness: 2.17±0.42 and 2.15±0.49; for sensory under-responsiveness: 1.98±0.47 and 1.96±0.16, respectively).

**Table 2:** Distribution of SMD subtypes within and between groups (N = 248).

SMD Status	Control Group (n = 204) N (%)	CRPS Group (n = 44) N (%)	Total
**Non-SMD**	178 (87.25)	29 (65.91)	207
sensory over-responsiveness	23 (11.27)	11(25.00)	34
sensory under-responsiveness	3 (1.47)	4 (9.09)	7

Non-SMD, no sensory modulation dysfunction

### 3.2 Distribution of SMD subtypes and their associations with CRPS Severity Score parameters in the CRPS group

CRPS Severity Score parameters were compared between SMD subtypes (i.e. sensory over-responsiveness; sensory under-responsiveness; and Non-SMD) within the CRPS group for each of the CSS variables: (8 self-report symptoms and 9 signs observed on examination by a clinician, a total of 17 items). No statistically significant differences were found between SMD subtypes (Kruskal-Wallis test p>0.05) in each of the CRPS Severity Score parameters tested. CRPS Severity Score parameters were also not found to be statistically significant different between males and females *p* = 1.00 (Fisher’s exact test).

### 3.3 SMD subtypes as risk factors for CRPS

SMD subtypes (sensory over-responsiveness [Yes/No] *vs*. Non-SMD, and sensory under-responsiveness [Yes/No] *vs*. Non-SMD] and age were found to be significantly related to CRPS in both univariate and multivariate models. Table 3 shows the adjusted odds ratios, 95% confidence intervals and level of significance when both variables were in the model. The odds ratio for age is presented in 5-year increments for ease of interpretation. For a patient with sensory over-responsiveness the risk of CRPS is 2.68 times higher than a subject without SMD, and for a subject with sensory under-responsiveness the risk is 8.21 times higher than a subject without SMD.

**Table 3:** Odds-ratios 95% Wald confidence limits and level of significance of risk factors for CRPS (N = 248).

Level/Variable	OR	95% Confidence Limits		p-value
sensory over-responsiveness **vs Non-SMD**	2.68	1.08	5.94	0.0261
sensory under-responsiveness **vs Non-SMD**	8.21	1.72	43.70	0.0079
**Age (per 5 years)**	1.34	1.04	1.74	0.0243

Non-SMD, no sensory modulation dysfunction

The regression model coefficients were then used to derive a risk score (or probability index) as P = e^Y^/ (1+e^Y^), where Y is a linear combination of the model coefficients. The risk score is presented on a scale of 0 to 1, where the higher the score the more likely the person is to have CRPS. From our model, we derived Y, the linear function of the regression coefficients to be:

Y=−3.4545+0.9556*(ifsensoryover-responsiveness)+2.1051*(ifsensoryunder-responsiveness)+0.0586*Age.

**Fig 1:**
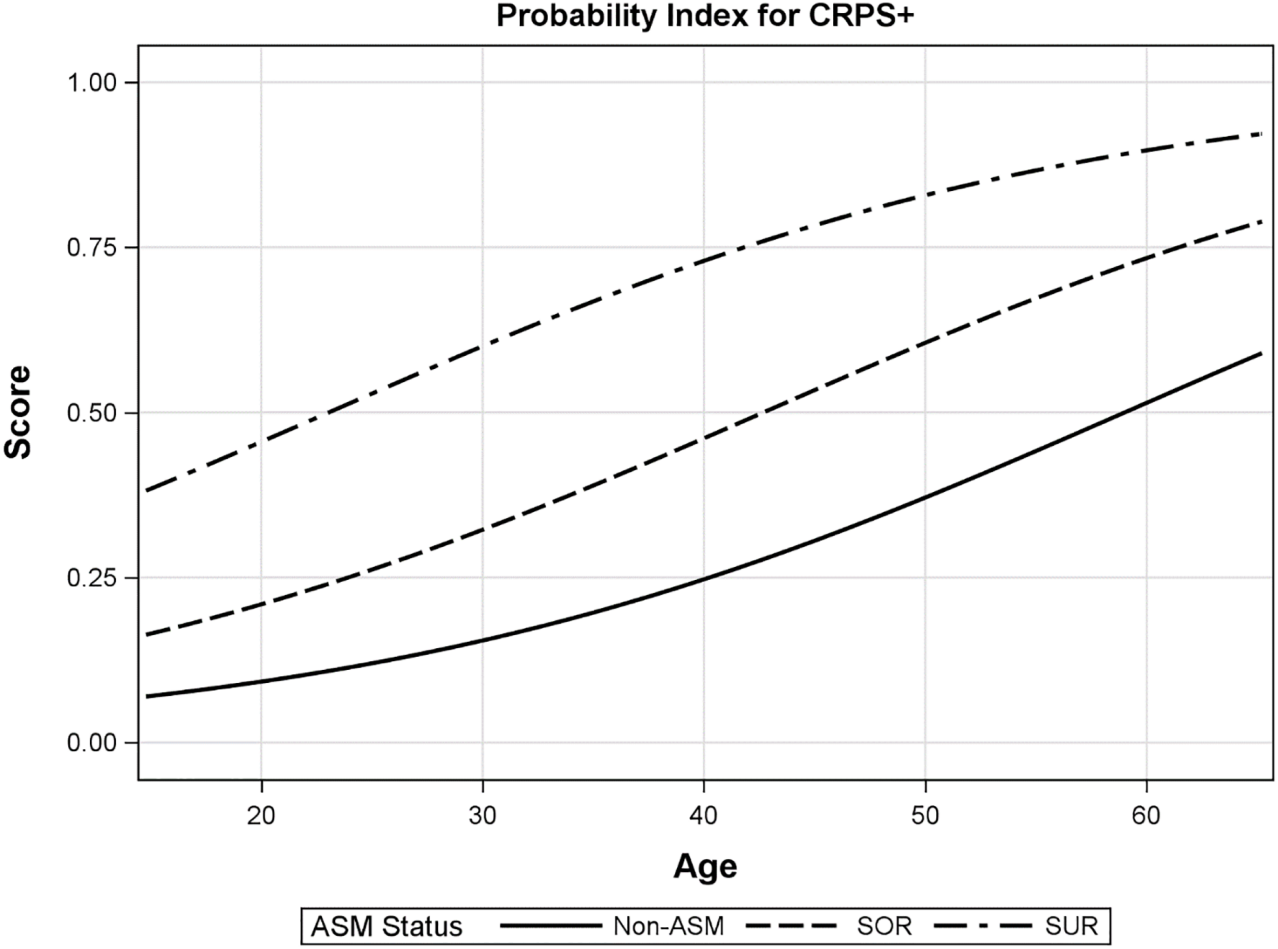
The risk probability for CRPS by SRQ-IS score. Non-SMD—no sensory modulation dysfunction; SOR—sensory over-responsiveness; SUR—sensory under-responsiveness. The risk index probability for CRPS takes into account the type of the sensory responsiveness i.e. over- or under-responsiveness (assessed by the SRQ-IS) and the person’s age (age is a continuous variable, so the fitted line can extend beyond the actual data).

This equation was depicted by an effect plot (Fig 1) to easily derive the score of an individual. Fig 1 demonstrates that while the risk score of a 30-year-old Non-SMD person to develop CRPS is about 10%, the score for the same age person with sensory under-responsiveness is nearly 60%. Moreover, a person who is 60 years old and Non-SMD has a score of 50%, while for the same age person with sensory under-responsiveness has a score of 90%. Thus, a person with sensory under-responsiveness is more likely to develop CRPS compared to NON-SMD person of the same age of 60.

## 4. Discussion

This study found that SMD together with age, contributed to explaining the presence of CRPS, thus, SMD and age may be potential risk factors for CRPS. Interestingly, being sensory under-responsive is associated with a higher likelihood of CRPS than being sensory over-responsive, or older in age. However, CRPS severity was not significantly different between SMD sub-types within patients with CRPS.

The leading sensory symptoms in CRPS are spontaneous and evoked pain, thus resulting in pain hypersensitivity (allodynia or/and hyperalgesia), but at the same time non-noxious sensory loss (hypoesthesia) also occurs [8, 47, 48]. In a study performed on 298 patients with less than 6 months duration of CRPS, results demonstrated a combination of sensitivity to painful stimuli and a moderate loss of sensitivity to non-painful stimuli. Importantly, the sensory loss was higher than previously documented, pointing at either excessive central inhibition or minimal nerve injury [48]. Sensory detection reduction together with spontaneous pain and allodynia or hyperalgesia can be either due to peripheral nerve lesion and successive spontaneous rostral afferent pathways activity [49] or be secondary to nociceptive stimuli, that is, pain-induced hypoesthesia [50, 51]. Further, a study performed on chronic CRPS patients also found warm and cool hypoaesthesia as well as hot and cold hyperalgesia, and importantly demonstrated a similar pattern of findings when the non-affected hand was tested. The authors suggest pathophysiological changes occurring sub-clinically in the non-affected hand, indicative of a central nervous system involvement [26]. Interestingly, a different perspective validates a predisposition of inter-individual differences serving as risk factors for developing CRPS, namely pre-existing pain mechanism alterations [47].

Indeed, endogenous pain modulation was tested in 27 patients with CRPS compared with age-matched healthy controls [47]. Findings revealed reduced adaptation to repetitive painful stimuli in patients with CRPS, both on the affected and unaffected hands when compared to healthy controls. Furthermore, patients with CRPS significantly demonstrated enhanced hyperalgesic areas on the affected side. Both findings were not correlated to individual disease symptoms. Therefore, the authors suggested that the changes could be a result of CRPS but also may point to pre-existing individual differences serving as a risk factor for CRPS development. Thus the presence of premorbid endogenous pain modulation alterations may also be assumed [47, 52].

Sensory over- and under- responsiveness are both manifestations of SMD. Testing pain perception in daily life contexts, outside the laboratory, indicated that SMD in otherwise healthy individuals co-occurs with daily pain sensitivity [28]. Interestingly, individuals with SMD have also demonstrated pain perception alterations in response to laboratory quantitative sensory testing of pain stimuli [41, 45]. Such alterations include hyper-sensitivity (hyperalgesia) and/or pain lingering sensations (pain after-sensation) compared to healthy controls, which are suggestive of compromised endogenous pain modulation [41]. Persistent activation of facilitation enhances neuronal sensitization in the dorsal horn and contributes to a state of chronic pain [53–55]. This co-occurrence of pain sensitivity (i.e. CRPS) and SMD could be explained by Mouraux et al’s (2011) study exploring whether there are indeed specific brain regions processing pain, referred to as the *pain matrix* [56]. The network of brain areas in the pain matrix revealed a significant BOLD fMRI response to stimuli, whether it was nociceptive or other. Specifically, nociceptive and non-nociceptive somatosensory stimuli evoked indifferent responses in S1 as well as in some portion of S2, indicating that part of the neural activities determining the BOLD response are evoked by non-nociceptive or nociceptive stimuli. These fMRI findings therefore propose that the network of regions, (i.e. the pain matrix), is likewise involved in nociceptive and non-nociceptive processing [56].

Though age was accounted as a risk factor in many studies [9, 17, 57, 58], it was not determined as a consistent potential risk factor for the onset of CRPS [11]. Females at any age pose a higher risk for the onset of CRPS [11]. However, findings are controversial and while literature shows that the risk for developing CRPS increases in postmenopausal women [9, 59–61], retrospective studies show a lower age onset, before the menopausal age [62, 63]. Importantly, a study of males in the armed forces showed that young males are vulnerable as well [64]. The present study adds to the evidence supporting age as a risk factor for CRPS. This finding is in line with the endogenous pain modulation deficiency that characterizes advancing age, in which the reduction in pain inhibition efficiency facilitates the effects of noxious stimuli on wide dynamic range neurons and elicits an increase in the pain experience [65].

This study has some limitation; as a cross sectional study, which included participants with and without CRPS we cannot verify development of CRPS in subjects with SMD. Moreover, under this study design, we cannot rule out that SMD could also be a consequence of, rather than a risk factor for CRPS. Thus, future studies should follow SMD and non-SMD subjects to verify that SMD and age can predict CRPS development. Another limitation may be the different size of the CRPS versus control groups, which lead to only 15 individuals (34.1%) versus 26 individuals (12.8%) with SMD, respectively. However, the small sample size in the CRPS group was sufficient to attain statistical significance since SMD is such a strong predictor of CRPS. Of note, that the estimated parameters (resulting in Fig 1) may be substantially altered in a larger study. Further, the CRPS patients this study reports on may form an atypical epidemiological group, as may be the case in a tertiary care hospital pain clinic, therefore our results might not necessarily be generalized to all CRPS patients. In addition, it should be noted that some of the patients received drugs for neuropathic pain or opioids, which might also have influenced their sensory modulation patterns. Lastly, Since CRPS is not the only chronic pain condition to manifest sensory abnormalities, further studies should verify whether the sensory pattern of under-responsiveness, found as the main potential risk factor for CRPS, is unique or may be generalized to other pain conditions. Of note, we recently found that migraineurs during pain-free phase demonstrate mostly sensory over-responsiveness [66], which may suggest that chronic pain conditions differ in their sensory abnormalities pattern.

### Conclusions

SMD is significantly associated with CRPS. Since SMD is easy and quick to identify, findings may add evidence supporting factors associated with CRPS, thus providing a new way for clinicians to assess a potential risk that may contribute to the early diagnosis of CRPS [11, 17]. To date no specific or sensitive clinical sign or symptom has been empirically found to be considered as a risk factor for the onset of CRPS [11]. However, evaluation by experienced clinicians accelerates the diagnosis [11, 67], and referring to pain management specialists and a multidisciplinary team (i.e. Occupational therapists; Physical therapists; psychologist) are related to better outcomes [68, 69]. Moseley et al. (2014) suggested that a numerical pain score of ≥ 5 in the first week following a fracture, could be clinically reasoned as a “red flag” for CRPS [17]. While this suggested sign could easily be identified by using a numerical pain scale, the current study provides a probability index for CRPS based on a self-report questionnaire, the Sensory Responsiveness Questionnaire—Intensity Scale [44]. The Sensory Responsiveness Questionnaire—Intensity Scale assesses responses to daily sensory events administered independently and does not require the presence of medical staff. Findings reveal that for a person with sensory under-responsiveness the risk of CRPS is 8.21 times higher than a person without SMD, and for a person with sensory over-responsiveness the risk is 2.68 times higher. Thus, including a CRPS risk factor into the clinical reasoning at the initial stage of intervention, or even before, may allow an early diagnosis and therefore a significant prognostic improvement. Future prospective studies would be beneficial to validate this cross-sectional study’s valuable findings defining a measurable risk factor for the debilitating condition of CRPS.

## Supporting information

S1 Table Data required for the analyses presented in this paper in an xlsx format. (XLSX)
